# P53. Effect of lymphodepletion and tumour on host and reconstituted regulatory T-cells in a model of murine melanoma

**DOI:** 10.1186/2051-1426-2-S2-P27

**Published:** 2014-03-12

**Authors:** J Kovács, P Rose, RA Hatz, H Winter, NK van den Engel

**Affiliations:** 1 Klinikum der Universität München-Großhadern, Klinik für Allgemeine Viszeral- Transplantations- Gefäß- und Thoraxchirurgie, München, Germany

## Background

Activation of tumour-specific T-cells is enhanced during homeostatic proliferation of lymphocytes in mice receiving *l*ymphodepletion, immune *r*econstitution and *a*ctive-*s*pecific *t*umour cell vaccination (LRAST). Nevertheless, the induction and the therapeutic efficacy of the tumor-specific T-cells is attenuated by immunoregulatory mechanisms such as regulatory T cells (Treg). Following LRAST, Treg can be derived from the host or the donor lymphocyte pool. Aim of the following study was to track the origin of the immune modulating Treg (host vs. donor) and to analyze their fate following LRAST.

## Material and methods

C57BL/6 mice were injected with D5 melanoma cells (5x10^4^ s.c.) to generate tumour bearing mice. After 3 days cyclophosphamide (200mg/kg i.p.) was injected to induce lymphopenia. On day 1 after lymphodepletion, mice were reconstituted with 2x10^7^ naïve spleen cells from a congenic strain of mice (C57BL/6-Ly5.1, 2x10^7^ cells i.v.) and vaccinated with irradiated, GM-CSF-producing D5G6 cells (1x10^7^ s.c.). These cells could be distinguished from the host T-cells based on their leukocyte surface molecule (host leukocytes: CD45.2^+^, donor leukocytes: CD45.1^+^). Mice without lymphodepletion served as a control. On day 3, 10 and 23 after lymphodepletion, cell populations were phenotypically analyzed by flow cytometry of splenocytes and blood cells. CD3^+^CD4^+^CD25^+^Foxp3^+^ cells were considered Treg; tumour specificity was controlled by IFN-g cytokine release assays.

## Results

In the lymphodepleted mice, flow cytometric analysis of splenocytes showed a decline in the host Treg between day 3 and day 10, followed by a recovery of the cells until day 23. In mice without lymphodepletion, host Treg remained at a constant level throughout the experiment. In contrast to that, we observed a steady increase over time in reconstituted Treg with a 5- to 8-fold increase from day 3 to day 23 in both lymphodepleted and not lymphodepleted mice. In LRAST-treated mice we were able to induce a higher frequency of tumour-specific T-cells compared to not lymphodepleted mice, as observed by tumor-specific cytokine release assays.

## Conclusion

Due to the proliferation of transferred Treg and increased levels of host Treg after lymphodepletion until day 23, the induction of tumor-specific T-cells might be limited. In future experiments we will determine whether the selective reduction of transferred Treg or the depletion of Treg with a specific anti-CD25 monoclonal antibody in the host will improve the induction of tumour-specific T cells and the therapeutic efficacy of active-specific tumour vaccination following LRAST.

